# Novel Quasi-Solid-State Electrolytes based on Electrospun Poly(vinylidene fluoride) Fiber Membranes for Highly Efficient and Stable Dye-Sensitized Solar Cells

**DOI:** 10.3390/nano9050783

**Published:** 2019-05-22

**Authors:** Fan Cheng, Ying Ou, Guoliang Liu, Li Zhao, Binghai Dong, Shimin Wang, Sheng Wen

**Affiliations:** 1 School of Materials Science and Engineering, Hubei University, Wuhan 430062, China; fancheng1123@stu.hubu.edu.cn (F.C.); lgl3685396@163.com (G.L.); zhaoli7376@163.com (L.Z.); dbh@hubu.edu.cn (B.D.); 2 College of Chemistry and Materials Science, Hubei Engineering University, Xiaogan 432000, China; ouying@whut.edu.cn; 3 Ministry-of-Education Key Laboratory for the Green Preparation and Application of Functional Materials, Hubei University, Wuhan 430062, China

**Keywords:** quasi-solid-state electrolyte, electrospun PVDF, high efficiency, stable, DSSCs

## Abstract

To obtain new highly efficient and stable quasi-solid dye-sensitized solar cells (QS-DSSCs) that can meet the requirements for the large-scale commercial application of solar cells, we have developed a novel quasi-solid-state electrolyte, based on an electrospun polyvinylidene fluoride (PVDF) membrane. The structure and properties of electrospun PVDF membranes were characterized by scanning electron microscopy (SEM), Brunauer–Emmett–Teller (BET), thermogravimetric (TG), and mechanical testing. The results indicate that the electrospun PVDF membrane has a three-dimensional network structure with extremely high porosity, which not only acts as a barrier to prevent electrolyte leakage but also provides a channel for the transmission of ions in the electrolyte, thereby effectively guaranteeing the high photoelectric conversion efficiency of the cells. The membrane was observed to withstand the conditions of hot-press (110 °C), and exhibited good thermal stability and mechanical strength, which are critical for the long-term stability and safety of the cells. The photovoltaic characteristics and stabilities of QS-DSSCs were compared with DSSCs based on an ionic liquid electrolyte (L-DSSC). QS-DSSCs with an 80 μm thick nanofiber electrolyte membrane showed a conversion efficiency of 8.63%, whereas an identical cell based on the corresponding ionic liquid electrolyte showed an efficiency of 9.30%. The stability test showed that, under indoor and outdoor conditions, after 390 h, the L-DSSCs failed. Meanwhile, the QS-DSSCs also maintained 84% and 77% of the original efficiency. The results show that, compared to the liquid electrolyte, the design of the quasi-solid electrolytes based on electrospun PVDF nanofiber membrane not only demonstrates the high conversion efficiency of DSSCs but also enhances the stability of the DSSCs, which provides the possibility for the fabrication of solar cells with higher efficiency and stability.

## 1. Introduction

With the increase in energy demand and environmental pollution in the last decade, global concerns about such problems have significantly spurred the technological endeavor of renewable and green energy. Among all the renewable energy, solar energy is considered to be one of the most promising sources of renewable energy, as it is rich in resources, clean, and pollution-free [1]. However, photovoltaic cells are the only devices that directly convert solar energy to electrical energy. The development of solar cells plays an important role in the area of solar energy conversion [1]. Since the first report by Oregan and Gratzel in 1991 [2], DSSCs have received massive attention owing to their relatively high energy conversion efficiencies along with their low projected production cost and simple fabrication process [3,4,5,6]. However, the traditional fabrication process of dye-sensitized solar cells (DSSCs) uses liquid electrolytes, which leads to long-term stability problems because of the leakage and volatility of liquid electrolytes [7,8]. To solve these problems, some solid electrolytes, such as inorganic *p*-type semiconductors [9,10,11,12,13] and organic hole-transport materials [14,15], have been applied in DSSCs. These solid-state electrolytes have a low evaporation rate compared to liquid electrolytes and thus enhance the lifetime of DSSCs. They also reduce the leakage problems and sealing cost of DSSCs. However, the efficiency of solid-state electrolytes is lower than liquid electrolytes due to poor penetration into mesoporous TiO_2_, lower ionic conductivity, low electron transfer from the dye molecules, and faster recombination. Therefore, for combining the best of both sides, the development of quasi-solid-state electrolytes, especially gel polymer electrolytes such as polyethylene glycol (PEG) [16], poly (methyl methacrylate-co-acrylonitrile) (PMMA-co-AN) [17], polyvinyl alcohol (PVA) [18], poly(ethyleneoxide)/polymethylmethacrylate (PEO/PMMA) [8], poly(ethyleneoxide)/polyethylene glycol (PEO/PEG) [7], and poly(ethyleneoxide)/poly(vinylidene fluoride-co-hexafluoropropylene) (PEO/PVDF-HFP) [19], has drawn much attention from the DSSC community. Although gel polymer electrolytes could resolve the problem of the leakage of liquid electrolyte to some extent, because of their complex preparation process, poor mechanical strength, and low thermal stability, there is a limitation when it comes to using them on a commercial scale [20,21]. To overcome these problems, another process, using polymer nanofiber membranes, has been developed [22,23,24,25].

For the synthesis of polymer nanofibers, techniques such as template synthesis [26,27], drawing [28], phase separation [29], and electrospinning [30] are generally employed. Of these techniques, electrospinning is considered to be the simplest and cheapest method for making ultrathin nanofibers.

Many electrospun polymeric nanofibrous membranes have been used to make quasi-solid electrolytes for DSSCs, but there are few studies on pure electrospun polyvinylidene fluoride (PVDF) fibrous membranes as an electrolyte material for DSSCs.

In an earlier study [31], we synthesized PVDF membranes by an electrospinning technique, and the electrospun PVDF nanofiber membranes were successfully applied to the composite polymer electrolytes of the fuel cells, which demonstrated excellent ionic conductivity. Based on the results of this study, it is predicted that the electrospun PVDF membrane can be used to prepare quasi-solid electrolyte membranes, and it is feasible to apply these to DSSCs, thus providing a new idea for the development of more efficient and stable DSSCs.

Therefore, in this study, electrospun PVDF membranes were prepared by a spinning technology to obtain quasi-solid electrolytes, and then the assembly of the DSSCs with the above-obtained quasi-solid electrolyte was conducted. The structure and properties of the electrospun PVDF membranes were characterized by SEM, BET, TG and mechanical testing, photoelectric conversion efficiency, and Electrochemical Impedance Spectroscopy (EIS) characteristics, along with the stability of the DSSCs, were tested. The observed results were then compared with the conventional ionic liquid electrolyte-based DSSCs.

## 2. Materials and Methods

### 2.1. Materials

Polyvinylidene fluoride (PVDF) (Kynar HSV 900, Arkema, Colombes, France), N, N-dimethylformamide (DMF), and acetone were purchased from Sinopharm Chemical Reagent Corp (Shanghai, China). A commercial ionic liquid electrolyte (DMII, I_2_, LiI, TBP, acetonitrile, NJU-AN-I), titania (TiO_2_) photoanode, Pt counter electrode, and Ruthenium dye (N719) were purchased from Kunshan Sunlaite Corp (Kunshan, China).

### 2.2. Preparation of Electrospun PVDF Membranes

**Figure 1:**
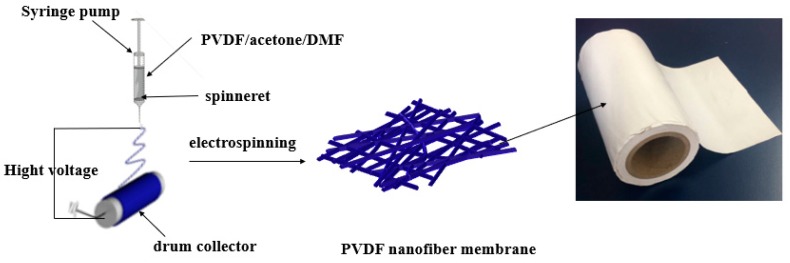
Schematic diagram illustrating the followed electrospinning process.

To prepare the electrospun PVDF membranes, PVDF powder was dissolved in an acetone/DMF (3:7, *w*/*w*) solution with magnetic stirring at room temperature for 24 h, and then underwent ultrasound treatment for 30 min to form a transparent homogeneous 16 wt % PVDF polymer solution. Figure 1 shows the preparation process of the electrospun PVDF membrane. A voltage of 15 kV was applied to the spinneret and drum collector to generate an electric field.

The flow of the polymer solution from the spinneret was adjusted to 0.5 mL/h using a syringe pump. The drum collector spun at a speed of 270 rpm, and the tip-to-collector distance was fixed at 20 cm. The membranes of different thicknesses were obtained by changing the diameter of the spinneret. The PVDF nanofiber membranes were placed in the vacuum oven at 60 °C for 10 h for later use.

### 2.3. Preparation of the Quasi-Solid-State Electrolyte

**Figure 2:**
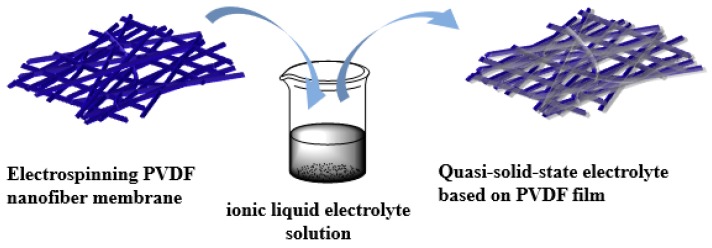
Schematic showing the process steps in the preparation of the quasi-solid-state electrolyte.

The electrospun PVDF fiber membrane was immersed in three drops of the ionic liquid electrolyte solution for about 30 min to ensure sufficient infiltration of the electrolyte to the nanofiber membrane. The excess solution on the membrane surface was then carefully wiped off with a filter paper to obtain the electrospun PVDF nanofiber membrane/ionic liquid electrolyte quasi-solid-state electrolyte. Figure 2 shows the process steps followed in the preparation of the quasi-solid-state electrolyte.

### 2.4. Fabrication of the Quasi-Solid-State DSSCs

**Figure 3:**
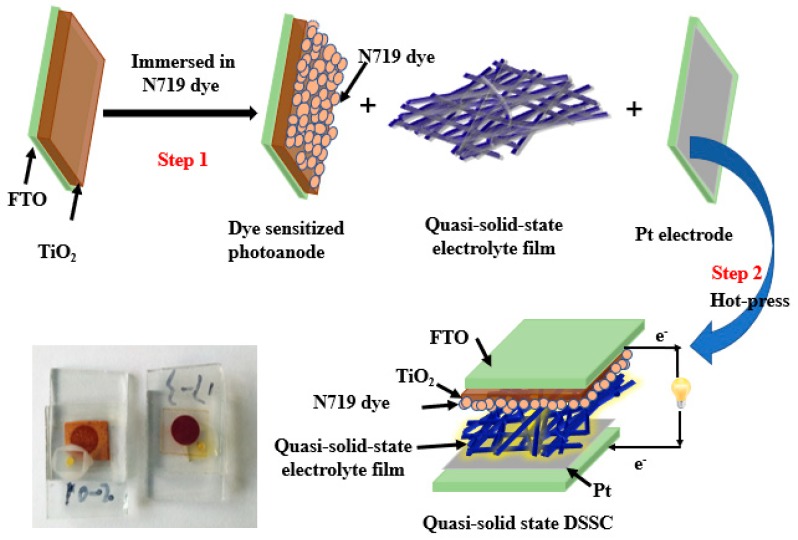
The schematic illustrates the steps involved in the manufacture of dye-sensitized solar cells (DSSCs) based on electrospun polyvinylidene fluoride (PVDF) nanofiber membranes.

The process followed to fabricate the quasi-solid-state DSSCs is schematically shown in Figure 3. DSSCs have a classic sandwich structure, consisting of a photosensitive photoanode, electrolyte, and an electrode. In this study, the commercial photoanode was placed into N719 dye solution and soaked for 12 h. After removal from the dye solution, the undissolved dye molecules on the surface of the photoanode were cleaned with anhydrous ethanol to obtain a photosensitive photoanode for later use. A commercial Pt counter electrode was selected along with the electrolytes, i.e., the ionic liquid electrolyte and a quasi-solid electrolyte, as prepared in step 2.3. The DSSC was assembled using a hot-press (110 °C). To compare the performance of the device, a DSSC was also fabricated with an ionic liquid electrolyte without PVDF. Each fabricated cell had an active area of 0.196 cm^2^.

### 2.5. Characterization

The morphology of electrospun PVDF membranes was characterized by field emission scanning electron microscopy (FE-SEM, TESCAN MIRA3). The uptake analysis measurement was measured by gravimetric method. Brunauer–Emmett–Teller (BET) surface area analysis was used to measure the specific surface area and pore structures of the membranes, using low temperature (77 K) nitrogen adsorption isotherms measured over a wide range of relative pressures, from 0.02 to 1. The adsorption measurements were performed on an ASAP2010 volumetric adsorption apparatus, and high purity nitrogen (99.9999%) was used in this experiment to avoid the effect of contamination. Before measurements, the samples were degassed at 100 °C for 6 h in the degas pot of the adsorption analyzer. Thermal stability of the electrospun membranes was carried out under a nitrogen flow using a thermogravimetric analyzer (TGA-STA 499F Instrument, Selber/Brvaria, Germany), where the samples were heated from 50 to 1000 °C at a heating rate of 10 °C min^−1^. Differential Scanning calorimeter (DSC) to investigate the melting behavior by a DSC-200 F3 instrument (NETZSCH Co., Selber/Bavaria, Germany). The mechanical properties of the films were tested using an electronic tensile machine (AGS-X, Shimadzu, Kyoto, Japan) to characterize the tensile strength. The tensile rate was set at 10 mm/min with a standard distance of 20 mm. The photovoltaic characteristics of the DSSCs were measured using a solar simulator (150 W simulator, Newport, Oriel 9408, 3A) under AM (air mass) 1.5 and 100 mW/cm^2^ of light intensity. The incident light intensities were calibrated using a reference cell. Th electrochemical impedance spectroscopy (EIS) measurements were tested using Zahner PP211, coupled with an IM6 electrochemical workstation in the frequency range of 10 mHz to 100 kHz and using an AC voltage signal of ± 20 V. These measurements were carried out under the illumination of 100 mW/cm^2^ using the same solar simulator. To further evaluate the stability of the DSSCs based on PVDF electrospun nanofiber membranes with a quasi-solid-state electrolyte and an ionic liquid electrolyte, the cells were placed under indoor (room temperature) and outdoor conditions (the aging box was used to simulate the harsh outdoor environment: temperature, 55 °C; humidity, 50%; irradiance of 1000 W/m^2^), and the electrochemical performance of the DSSCs was characterized using a solar simulator (150 W simulator, Newport, Oriel 9408, 3A) under AM 1.5 and 100 mW/cm^2^ of light intensity. The incident light intensities were calibrated using a reference cell at regular intervals.

## 3. Results and Discussion

For consistency, unless otherwise stated, PVDF-1 and PVDF-2 correspond to electrospun PVDF nanofiber films with a thickness of 60 μm and 80 μm, respectively. The quasi-solid DSSCs based on the electrospun PVDF nanofiber membranes with a thickness of 60 μm and 80 μm are labeled QS-DSSC-1 and QS-DSSC-2, respectively, and the ionic liquid electrolyte DSSC is labeled L-DSSC.

### 3.1. Electrospun PVDF Membrane

**Figure 4:**
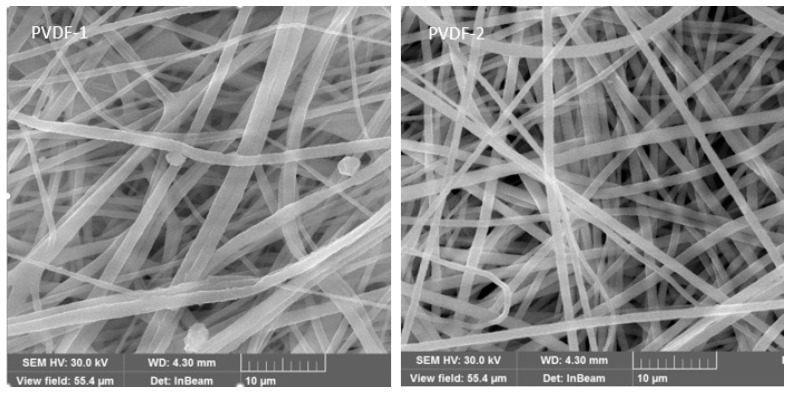
The SEM images of the electrospun PVDF membranes.

The morphology and structure of the electrospun PVDF membranes with a thickness of 60 μm and 80 μm were characterized by SEM, as shown in Figure 4. It is clear from these images that the PVDF membrane has a three-dimensional network structure with extremely high porosity, consisting of thin fibers, which is an effective barrier to prevent the leakage of the electrolyte. The fiber diameters of the electrospun PVDF nanofiber membranes with a thickness of 60 μm and 80 μm are 500–1200 nm and 500–1500 nm, respectively. The well-interconnected porous structure and highly tortuous pores formed by the smooth nanofibers could increase the ability of the membrane to trap the liquid electrolyte and facilitate the transport of ions in the inner space of the membrane, which can effectively guarantee the high photoelectric conversion efficiency of the cells.

In order to characterize the absorption performance of the membrane to the electrolyte solution, the membrane weight before soaking the electrolyte was denoted as *m*_0_, the membrane was immersed in the electrolyte, and the membrane was removed after 30 min to wipe off the excess solution on the surface. The weight was denoted as *m*_w_, the absorbance calculated, three parallel tests were performed, and the average calculated. The calculation formula is as follows:

(1) $$\mathrm{uptake}\;\mathrm{rate}(\%)=\frac{m_{w}-m_{0}}{m_{w}}\times 100\%$$

As can be seen from the Table 1, the absorbance of the PVDF membrane with a thickness of 80 μm is higher than that of the 60 μm membrane.

**Figure 5:**
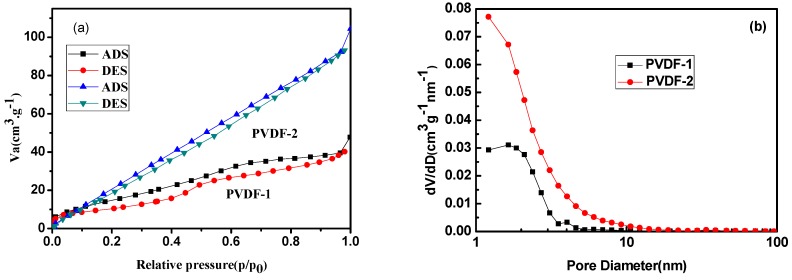
(**a**) Nitrogen adsorption–desorption isotherm, (**b**) pore size distribution plots of PVDF fiber membranes.

**Figure 6:**
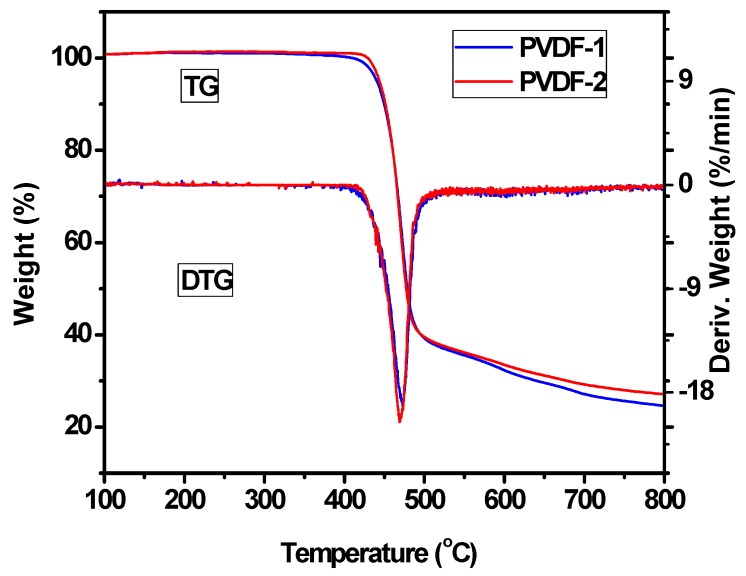
TG and DTG curves of the electrospun PVDF membranes.

To further investigate the specific surface area and porous nature of the electrospun PVDF membranes, Brunauer–Emmett–Teller (BET) gas sorptometry measurements were conducted. Figure 5 and Figure 6 show the nitrogen adsorption–desorption isotherms and the pore size distribution plots of PVDF membranes. It can be seen that all samples exhibited a type II sorption isotherm, with a hysteresis of type H4, which is characteristic of mesoporous materials [32]. The pore size distribution, derived from the desorption data and calculated from the Barrett-Joyne-Halenda (BJH) model, shows that most of the pore sizes of the electrospun PVDF films with a thickness of 60 μm and 80 μm were between 1 and 5 nm, and the average pore sizes were 4.66 nm and 4.20 nm, respectively, which confirms that the fibers are mesoporous materials. The obtained BET specific surface area, total pore volume, and mean pore diameter of the nanofiber mats for this study are listed in Table 2. It can be seen that the specific surface area increases from 60.68 to 148.10 m^2^g^−1^ as the thickness increased from 60 to 80 μm. In the process of electrospinning, the solvent with the larger diameter volatilizes more slowly, and more pores are formed in the process, resulting in a larger specific surface area, which demonstrates that the electrospun PVDF membrane with a thickness of 80 μm has a stronger surface adsorption capacity, which is essential to obtain higher efficiency.

**Figure 7:**
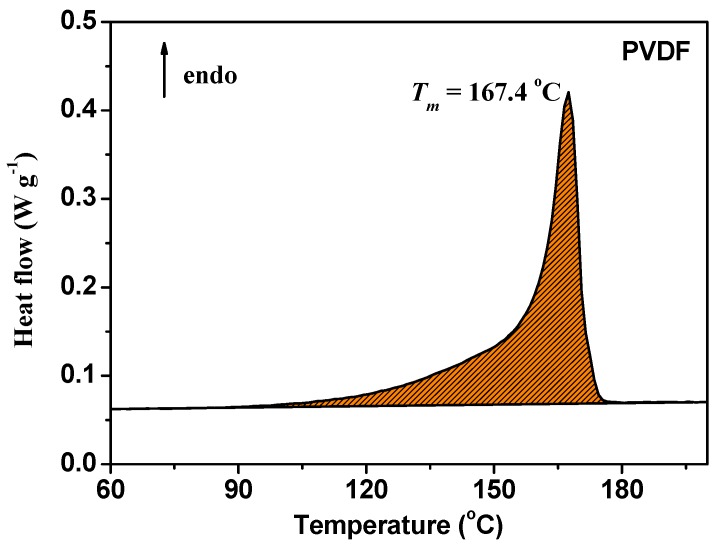
DSC curve of the electrospun PVDF membrane.

The thermal stability of electrospun PVDF fiber membranes is a crucial property for their long-time use during the operation of DSSCs. Thermal gravity analysis (TGA) is a useful technique to evaluate the changes in the mass of PVDF fiber membranes and their thermal stability. From the TG and differential thermal gravity (DTG) curves, as shown in Figure 6, there is only a single step weight loss, which starts at about 420 °C and ends at about 520 °C and corresponds to the degradation of the main chains of PVDF [31,32]. After degradation, the mass of the PVDF fiber membrane is reduced by 80%. The thermal degradation temperature of the material is very high, indicating that the material has excellent thermal stability. Figure 7 shows the DSC curve of the electrospun PVDF fiber membrane. It can be seen from the figure that the melting temperature (T_m_) of PVDF is 167.4 °C, and therefore the electrospun PVDF fiber membrane can withstand a temperature of 110 °C in the hot-pressing process.

**Figure 8:**
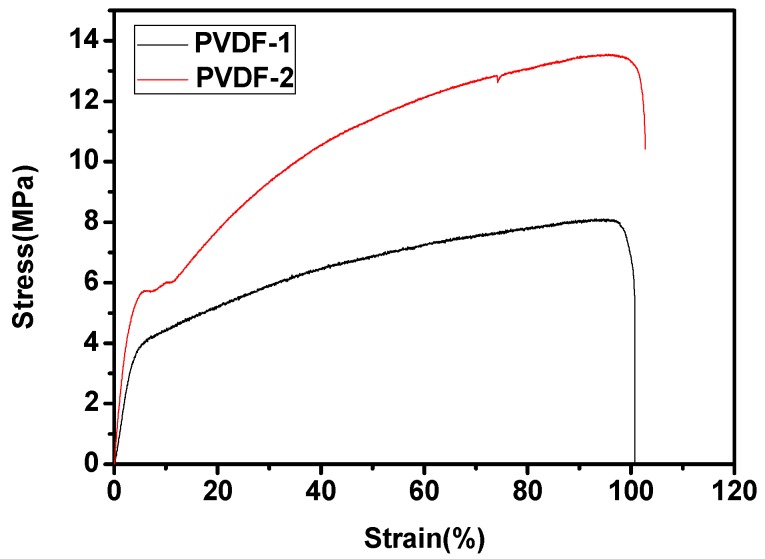
Stress–strain curves of the electrospun PVDF membranes.

Figure 8 shows the stress–strain curves of the electrospun PVDF fiber membranes. As can be seen from this figure, the fiber membranes with different thicknesses show distinct ductile fracture characteristics; when the thickness of films increased from 60 to 80 μm the toughness of the films was notably improved. Compared with PVDF-1, the fracture strength of PVDF-2 was higher, reaching 13.5 MPa. However, the change in thickness did not cause a significant alternation in elongation at the break. This indicates that the strength and toughness of electrospun PVDF membranes are improved when the thickness is increased. The excellent strength and toughness of the electrospun PVDF film indicates that the film has robust mechanical properties, which is conducive to the encapsulation of the cells. Therefore, after electrolyte immersion and thermal encapsulation, the films maintain good morphology and structure.

### 3.2. Photovoltaic Performance

**Figure 9:**
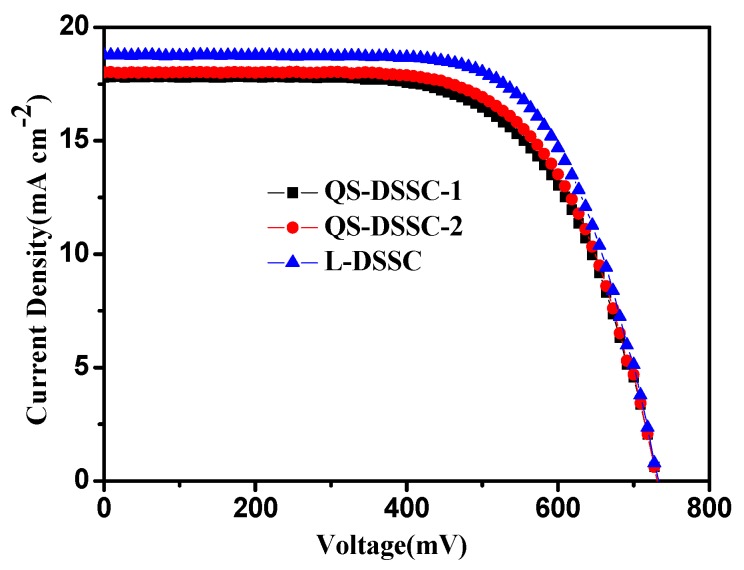
Photocurrent density–voltage (J–V) curves of the DSSCs with quasi-solid-state electrolytes, based on the electrospun PVDF membranes and ionic liquid electrolytes.

Figure 9 shows the photocurrent density–voltage (J-V) curves for solar cells, based on electrospun PVDF nanofiber membrane electrolytes and ionic liquid electrolytes. The photovoltaic characteristics of the fabricated DSSCs are summarized in Table 2.

Based on Figure 9 and Table 3, it can be seen that the cells assembled by ionic liquid electrolyte had the highest efficiency (η)—reaching 9.3%--and their observed open circuit voltage (V_OC_), short circuit current density (J_SC_), and fill factor (FF) were 0.73 V, 18.79 mA cm^−2^, and 68%, respectively. Although the efficiencies of QS-DSSCs are lower than those of L-DSSC, they are much higher than those of cells using other polymers, as reported in many other studies [22,23,24,25]. QS-DSSC-2 has an η of 8.63%, V_OC_ of 0.73 V, J_SC_ of 18.01 mAcm^−2^, and FF of 66%, which is outstanding compared to QS-DSSC-1. The efficiency of the QS-DSSCs is slightly lower than the L-DSSC, mainly due to the electrospun fiber membrane, which has an interwoven network structure that hinders the mobility of the iodine ion in the I^−^/I_3_^−^ between the TiO_2_ photosensitive electrode and Pt electrode. Besides this, it limits the regeneration rate of the dye, leading to the reduction of J_SC_, and resulting in a decrease in the photoelectric conversion efficiency. When the thickness of the film increased from 60 to 80 μm, the efficiency of the cell also increased accordingly. With a thickness of 60 μm, the specific surface area and pore volume of the electrospun membrane were low (from BET results), which resulted in a low uptake of electrolyte solution for the PVDF membrane. The low uptake of the electrolyte caused less contact with the dye-sensitized TiO_2_ electrode, which led to a low J_SC_ value. When the thickness of the membrane increased to 80 μm, the number of pores and the pore volume increased, which allowed the membrane to uptake more electrolyte solution. The higher amount of electrolyte solution increased the penetration into the nanoporous structure of TiO_2_ and led to a higher regeneration of dye, which enabled the cells to provide a higher photocurrent density.

**Figure 10:**
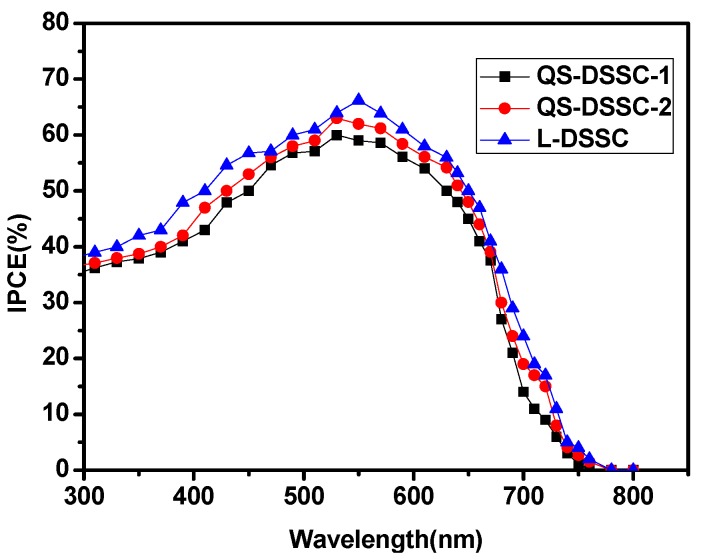
IPCE spectra of the DSSCs with quasi-solid-state electrolytes based on the electrospun PVDF nanofiber membranes and ionic liquid electrolyte.

The photochemical properties of polymer gel electrolytes were studied by monochromatic incident photon-to-electron conversion efficiency (IPCE) spectroscopy. Figure 10 shows the IPCE spectrum of the DSSCs with quasi-solid-state electrolytes based on the electrospun PVDF nanofiber membranes and ionic liquid electrolyte. The values of IPCE are proportional to the values of J_SC_. This result supports the J–V characteristics of the DSSCs with quasi-solid-state electrolytes based on the electrospun PVDF nanofiber membranes and ionic liquid electrolytes.

**Figure 11:**
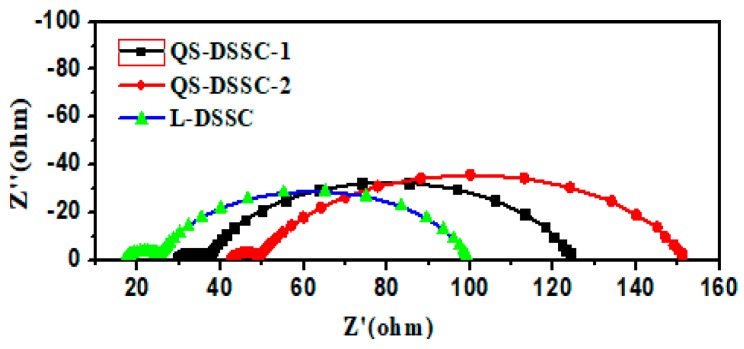
Nyquist plots of the DSSCs with quasi-solid-state electrolytes and ionic liquid electrolytes.

**Figure 12:**
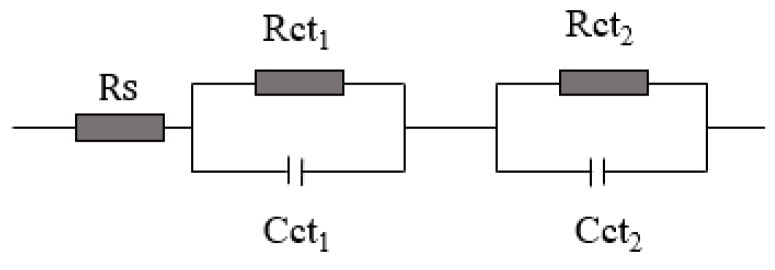
The equivalent circuit of the DSSC devices.

To understand the effect of fiber in the matrix, the interfacial charge transfer resistances of the three types of DSSCs were investigated through EIS measurements. The Nyquist plots of the DSSCs with three different electrolyte systems and the equivalent circuit of the DSSCs are shown in Figure 11 and Figure 12, respectively.

In the Nyquist plots, the first small semicircle corresponds to the charge transfer resistance of the Pt/electrolyte interface (Rct_1_) and the second bigger semicircle corresponds to the charge transfer resistance of the TiO_2_/electrolyte interface (Rct_2_).

Table 4 summarizes the calculated values of the series resistance (R_S_), the charge transfer resistance of the Pt/electrolyte interface (Rct_1_), and the charge transfer resistance of the TiO_2_/electrolyte interface (Rct_2_) for the three types of fabricated DSSCs.

These results show that the Rct_1_ of the QS-DSSCs is similar to the Rct_1_ of the L-DSSC. However, the R_S_ and Rct_2_ of the QS-DSSCs are higher than the R_S_ and Rct_2_ of the L-DSSC. This show that the DSSC using a fiber membrane has a higher resistance. The main reason for these changes in resistance values may be that the introduction of an electrospun PVDF fiber film increases the series resistance (R_S_) and the resistance of charge transfer between the interface of the TiO_2_/dye/electrolyte, which in turn increases with an increase in the thickness, since the use of a fiber matrix reduces the ionic mobility due to the blocking effect, compared to the ionic liquid electrolyte cell. As a result, the efficiency (η) of the DSSC using an electrospun PVDF film showed a low value.

### 3.3. Stability of the Quasi-Solid-State DSSCs

**Figure 13:**
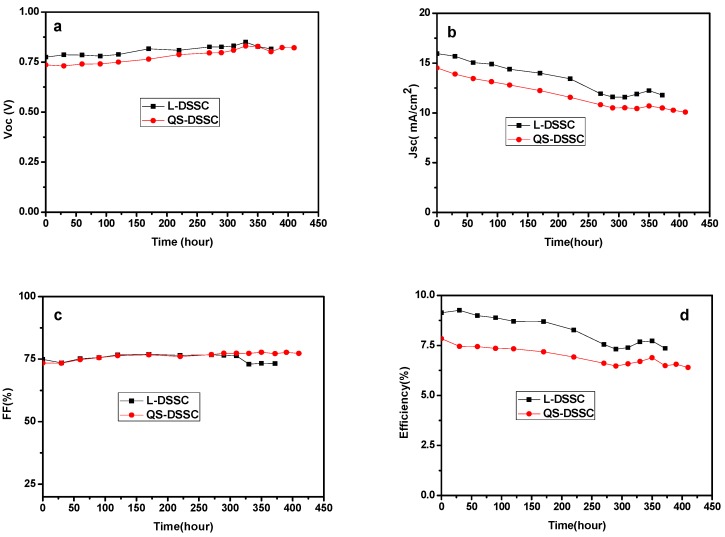
Normalized photoelectric performance of the QS-DSSC and L-DSSC versus time under indoor conditions: (**a**) V_OC_, (**b**) J_SC_, (**c**) FF, and (**d**) η.

**Figure 14:**
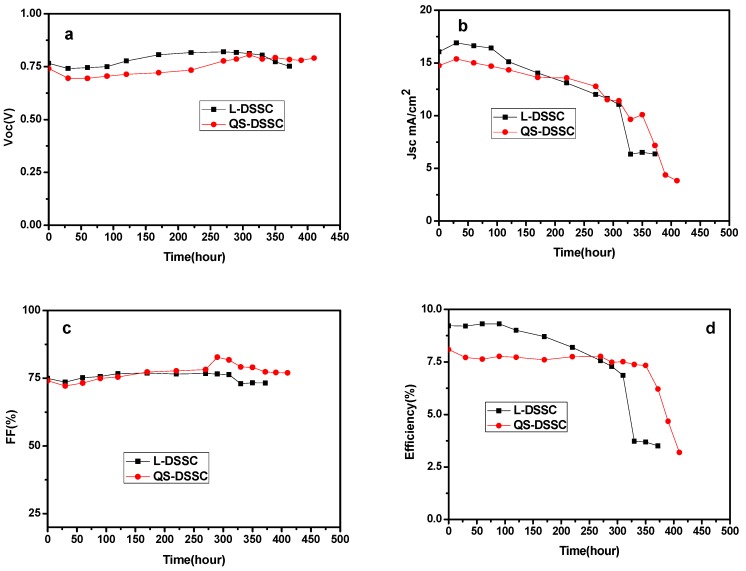
Normalized photoelectric performance of the QS-DSSC and L-DSSC versus time under outdoor conditions: (**a**) V_OC_, (**b**) J_SC_, (**c**) FF, (**d**) η.

Figure 13 and Figure 14 show the variation curves of four performance parameters (V_OC_, J_SC_, FF, and η) of the cells with time, under indoor and outdoor conditions, respectively. The comprehensive performance of the membrane with a thickness of 80 μm was significantly better than that of the 60 μm membrane. Therefore, in the stability test, the quasi-solid DSSC composed of 80 μm film was selected for comparison with the liquid DSSC. As can be seen from Figure 12, compared with L-DSSC, the Voc and FF values of the QS-DSSC increased. The Jsc and η values of the QS-DSSC decreased, but the changes in the values of the four parameters of the QS-DSSC over time are relatively smaller than that of the L-DSSC, indicating that the QS-DSSC is more stable than the L-DSSC as a whole. Based on Figure 13b,d, under the same sealing conditions, the reduction of J_SC_ is caused by the volatilization of the electrolyte, which also indicates that the electrospun PVDF fiber membrane can prevent the volatilization of the electrolyte more effectively. After 390 h, the L-DSSC without the PVDF electrospun film failed, and the QS-DSSC retained 84% of its initial efficiency. These results indicate that the quasi-solid electrolyte based on an electrospun PVDF nanofiber membrane is more stable than the liquid electrolyte, which ultimately leads to better stability of the QS-DSSC than the L-DSSC.

To further compare the stability of the QS-DSSCs and L-DSSCs, stability testing was carried out under outdoor conditions. As can be seen from Figure 14b, the J_SC_ of the L-DSSC began to decline significantly after 310 h, while that of the QS-DSSCs began to decline significantly after 372 h of operation. Under the same sealing conditions, the decline of the J_SC_ may be caused by the volatilization or leakage of the electrolyte. Based on the observations, the efficiency of the L-DSSC, which runs up to 330 h, is only 40% of its original efficiency, while the QS-DSSCs maintain 91% of their original efficiency. At 390 h, the L-DSSC failed, whereas the QS-DSSCs retained 77% of their initial efficiency. Through the comparison of the obtained data, it can be found that QS-DSSCs are more stable than L-DSSCs, since the liquid electrolyte in the outdoor environment is quite volatile and leaky, and following the preparation of the electrospun PVDF nanofiber membrane of the quasi-solid electrolyte effectively solves this problem. The three-dimensional network structure of the electrospun fiber membrane is a barrier to prevent the leakage of the liquid electrolyte, and at the same time, the transfer between holes provides channels for the ions.

## 4. Conclusions

In this study, electrospun PVDF fiber membranes were successfully applied to DSSCs. The electrospun PVDF membrane has a three-dimensional network structure with extremely high porosity. The membrane can withstand the conditions of the hot-press (110 °C), which showed an excellent absorption property for the ionic liquid electrolyte, good thermal stability, and mechanical strength. DSSCs with quasi-solid-state electrolyte showed a light-to-electricity conversion efficiency of 8.63%, which is very close to the performance of DSSCs fabricated using an ionic liquid electrolyte. Moreover, the stability tests showed that under both indoor and outdoor conditions, the stability of the QS-DSSCs was higher than the L-DSSCs. These results suggest that the electrospun PVDF fiber membranes could serve as new high-performance quasi-solid electrolyte materials in DSSCs.

## Figures and Tables

**Table 1:** The absorption performance of PVDF electrospun nanofiber membranes.

Samples	Uptake Rate1 (%)	Uptake Rate2 (%)	Uptake Rate3 (%)	Average Uptake Rate (%)
PVDF-1	57	59	56	57.3
PVDF-2	69	65	68	67.3

**Table 2:** The Brunauer–Emmett–Teller (BET) specific surface area, total pore volume, and mean pore diameter of PVDF electrospun fiber membranes.

Samples	Specific Surface Area (m^2^·g^−1^)	Pore Volume (cm^3^·g^−1^)	Mean Pore Diameter (nm)
PVDF-1	60.68	0.07	4.66
PVDF-2	148.10	0.16	4.20

**Table 3:** Photovoltaic parameters of the DSSCs with electrospun PVDF membrane electrolytes and ionic liquid electrolytes.

Samples	η (%)	V_OC_ (V)	J_SC_ (mA.cm^−2^)	FF (%)
QS-DSSC-1	8.36	0.73	17.79	64
QS-DSSC-2	8.63	0.73	18.01	66
L-DSSC	9.30	0.73	18.76	68

**Table 4:** The series resistance (R_S_) and charge transfer resistance of the Pt/electrolyte interface (Rct_1_), and the charge transfer resistance of the TiO_2_/electrolyte interface (Rct_2_) in DSSCs using three types of electrolytes.

Samples	R_S_	Rct_1_	Rct_2_
QS-DSSC-1	29.49	7.232	87.84
QS-DSSC-2	42.37	6.622	103.1
L-DSSC	16.95	8.225	74.33

## References

[1] Su’ait M.S., Rahman M.Y.A., Ahmad A. (2015). Review on polymer electrolyte in dye-sensitized solar cells (DSSCs). Sol. Energy.

[2] O’Regan B., Gratzel M. (1991). A low-cost, high-efficiency solar cell based on dye-sensitized colloidal TiO_2_ films. Nature.

[3] Mohan K., Bora A., Nath B.C., Gogoi P., Saikia B.J., Dolui S.K. (2016). A highly stable and efficient quasi solid state dye sensitized solar cell based on Polymethylmethacrylate (PMMA)/Polyaniline Nanotube (PANI-NT) gel electrolyte. Electrochim. Acta.

[4] Yuan S.S., Tang Q.W., He B.L., Yang P.Z. (2014). Efficient quasi-solid-state dye-sensitized solar cells employing polyaniline and polypyrrole incorporated microporous conducting gel electrolytes. J. Power Sources.

[5] Lee R.H., Cheng T.F., Chang J.W., Ho J.H. (2011). Enhanced photovoltaic performance of quasi-solid-state dye-sensitized solar cells via incorporating quaternized ammonium iodide-containing conjugated polymer into PEO gel electrolytes. Colloid Polym. Sci..

[6] Li Q.H., Chen X.X., Tang Q.W., Cai H.Y., Qin Y.C., He B.L., Li M.J., Jin S.Y., Liu Z.C. (2014). Enhanced photovoltaic performances of quasi-solid-state dye sensitized solar cells using a novel conducting gel electrolyte. J. Power Sources.

[7] Anantharaj G., Joseph J., Selvaraj M., Jeyakumar D. (2015). Fabrication of stable dye sensitized solar cell with gel electrolytes using poly (ethylene oxide)-poly (ethylene glycol). Electrochim. Acta.

[8] Aram E., Ehsani M., Khonakdar H.A. (2015). Improvement of ionic conductivity and performance of quasi-solid-state dye sensitized solar cell using PEO/PMMA gel electrolyte. Thermochim. Acta.

[9] Tennakone K., Kumara G.R.R.A., Kumarasinghe A.R., Wijayantha K.G.U., Sirimanne P.M. (1995). A dye-sensitized nano-porous solid-state photovoltaic cell. Semicond. Sci. Technol..

[10] O’Regan B., Schwartz D.T. (1995). Efficient photo-hole injection from adsorbed cyanine dyes into electrodeposited copper (I) thiocyanate thin films. Chem. Mater..

[11] Kumara G.R.R., Konno A., Senadeera G.K.R., Jayaweera P.V.V., De Silva D.B.R.A., Tennakone K. (2001). Dye-sensitized solar cell with the hole collector p-CuSCN deposited from a solution in n-propyl sulphide. Sol. Energy Mater. Sol. Cells..

[12] O’Regan B., Schwartz D.T. (1998). Large enhancement in photocurrent efficiency caused by UV illumination of the dye-sensitized heterojunction TiO_2_/RuLL NCS/CuSCN: Initiation and potential mechanisms. Chem. Mater..

[13] Kavan L., Saygili Y., Freitag M., Zakeeruddin S.M., Hagfeldt A., Grätzel M. (2017). Electrochemical properties of Cu(II/I)-based redox mediators for dye-sensitized solar cells. Electrochim. Acta.

[14] Schon J.H., Kloc C., Bucher E., Batlogg B. (2000). Efficient organic photovoltaic diodes based on doped pentacene. Nature.

[15] Shaheen S.E., Brabec C.J., Sariciftci N.S., Padinger F., Fromherz T., Hummelen J.C. (2001). 2.5% efficient organic plastic solar cells. Appl. Phys. Lett..

[16] Gong J., Sumathy K., Liang J. (2012). Polymer electrolyte based on polyethylene glycol for quasi-solid state dye sensitized solar cells. Renew. Energy.

[17] Lan Z., Wu J.H., Lin J.M., Huang M.L. (2011). Quasi-solid-state dye-sensitized solar cells containing P (MMA-co-AN)-based polymeric gel electrolyte. Polym. Adv. Technol..

[18] Arof A.K., Naeem M., Hameed F., Jayasundara W.J.M.J.S.R., Careem M.A., Teo L.P., Buraidah M.H. (2014). Quasi solid state dye-sensitized solar cells based on polyvinyl alcohol (PVA) electrolytes containing I^-^/I_3_^-^ redox couple. Opt. Quantum Electron..

[19] Cui Y.Z., Zhang J., Zhang X.N., Feng J.W., Hong Y., Zhu Y.J. (2012). High performance quasi-solid-state dye-sensitized solar cells based on acetamide-modified polymer electrolytes. Org. Electron..

[20] Zhao J.X., Jo S.G., Kim D.W. (2014). Photovoltaic performance of dye-sensitized solar cells assembled with electrospun polyacrylonitrile/silica-based fibrous composite membranes. Electrochim. Acta.

[21] Park S.H., Won D.H., Choi H.J., Hwang W.P., Jang S.I., Kim J.H., Jeong S.H., Kim J.U., Lee J.K., Kim M.R. (2011). Dye-sensitized solar cells based on electrospun polymer blends as electrolytes. Sol. Energy Mater. Sol. Cells.

[22] Dissanayake M.A.K.L., Divarathne H.K.D.W.M.N.R., Thotawatthage C.A., Dissanayake C.B., Senadeera G.K.R., Bandara B.M.R. (2014). Dye-sensitized solar cells based on electrospun polyacrylonitrile (PAN) nanofibre membrane gel electrolyte. Electrochim. Acta.

[23] Vijayakumar E., Subramania A., Fei Z.F., Dyson P.J. (2015). Effect of 1-butyl-3-methylimidazolium iodide containing electrospun poly (vinylidene fluoride-*co*-hexafluoropropylene) membrane electrolyte on the photovoltaic performance of dye-sensitized solar cells. J. Appl. Polym. Sci..

[24] Kim J.U., Park S.H., Choi H.J., Lee W.K., Lee J.K., Kim M.R. (2009). Effect of electrolyte in electrospun poly(vinylidene fluoride-*co*-hexafluoropropylene) nanofibers on dye-sensitized solar cells. Sol. Energy Matrt. Sol. Cells.

[25] Park S.H., Choi H.J., Lee S.B., Lee S.M., Cho S.E., Kim K.H., Kim Y.K., Kim M.R., Lee J.K. (2011). Fabrications and photovoltaic properties of dye-sensitized solar cells with electrospun poly (vinyl alcohol) nanofibers containing Ag nanoparticles. Macromol. Res..

[26] Feng L., Li S., Li H., Zhai J., Song Y., Jiang L., Zhu D. (2002). Super-hydrophobic surface of aligned polyacrylonitrile nanofibers. Angew. Chem. Int. Ed..

[27] Martin C.R. (1996). Membrane-based synthesis of nanomaterials. Chem. Mater..

[28] Ondarcuhu T., Joachim C. (1998). Drawing a single nanofibre over hundreds of microns. Europhys. Lett..

[29] Ma P.X., Zhang R. (1999). Poly (alpha-hydroxyl acids)/hydroxyapatite porous composites for bone-tissue engineering. I. Preparation and morphology. J. Biomed. Mater. Res..

[30] Deitzel J.M., Kleinmeyer J., Harris D., Tan N.C.B. (2001). The effect of processing variables on the morphology of electrospun nanofibers and textiles. Polymer.

[31] Gong C.L., Liu H., Zhang B.Q., Wang G.J., Cheng F., Zheng G.W., Wen S., Xue Z.G., Xie X.L. (2017). High level of solid superacid coated poly (vinylidene fluoride) electrospun nanofiber composite polymer electrolyte membranes. J. Membr. Sci..

[32] Lolla D., Lolla M., Abutale A., Shin H.U., Reneker D.H., Chase G.G. (2016). Fabrication, polarization of electrospun polyvinylidene fluoride electret fibers and effect on capturing nanoscale solid aerosols. Materials.

